# Atherosclerosis Specific Features in Chronic Kidney Disease (CKD)

**DOI:** 10.3390/biomedicines10092094

**Published:** 2022-08-27

**Authors:** Anastasia V. Poznyak, Nikolay K. Sadykhov, Andrey G. Kartuesov, Evgeny E. Borisov, Vasily N. Sukhorukov, Alexander N. Orekhov

**Affiliations:** 1 Institute for Atherosclerosis Research, Osennyaya 4-1-207, 121609 Moscow, Russia; 2 Laboratory of Angiopathology, Institute of General Pathology and Pathophysiology, 8 Baltiiskaya Street, 125315 Moscow, Russia; 3 Petrovsky National Research Centre of Surgery, Abrikosovsky Lane, 119991 Moscow, Russia

**Keywords:** atherosclerosis, CKD, chronic kidney disease

## Abstract

Atherosclerosis is the major cause of cardiovascular disease, leading to a high mortality rate worldwide. Several risk factors are known to favor atherogenesis, among which are high blood pressure, smoking, diabetes mellitus, and others. Chronic kidney disease is another serious health problem associated with significant health care costs, morbidity, and mortality. Chronic kidney disease shares several risk factors with atherosclerosis and cardiovascular diseases, such as hypertension and diabetes mellitus. Additional risk factors for cardiovascular disease development should be considered in patients with chronic kidney disease. Interestingly, patients suffering from chronic kidney disease are more prone to cardiovascular problems than the general population. Moreover, chronic kidney disease is characterized by an increased atherosclerotic burden from the very early stages. The purpose of this review was to summarize data on atherosclerosis in chronic kidney disease, highlighting the specific features of the disease combination.

## 1. Introduction

The traditional risk factors for cardiovascular morbidity and mortality are not valuable enough when we are talking about chronic kidney disease (CKD) patients. Thus, we can suggest that some supplementary mechanisms and pathogenic processes can participate in emerging CKD-related risk factors [1]. Unfortunately, these specific mechanisms are not sufficiently investigated. On the other hand, very few genes were differentially expressed in healthy arteries from advanced patients with CKD compared to normal renal function individuals. In particular, only 23 genes consistently modulated in vascular smooth muscle cells (VSMCs) were downregulated, and 8 of them were upregulated. Additionally, gene expression was not always accompanied by parallel changes in cellular protein content. For example, both the mRNA expression and the protein content of the alpha subunit of the hypoxia-inducible factor 3 increased as a consequence of uremia, whereas vimentin content increased with a decreased expression of its mRNA [2].

In patients with CKD, three essential factors of cardiovascular disease (CVD) risk reduction can be noted: (1) accurate diagnosis of CKD, (2) recognition of the elevated risk of CVD, and (3) early identification and modifiable risk factors management [3].

## 2. Chronic Kidney Disease (CKD)

The syndrome of a chronicle change in the structure or function of one or both kidneys, which subsequently affects the health, is known as chronic kidney disease (CKD) [4]. Tumors, cysts, malformations, and atrophy, which are noticeable during visualization, are examples of structural abnormalities. Despite this, in children, edema, hypertension, changes in the volume or quality of urine, as well as growth retardation may be signs of alteration of kidney function. Usually, such changes can be detected by increased levels of creatinine, cystatin C, or urea nitrogen in the blood serum. Kidney fibrosis is one of the common pathological manifestations of CKD regardless of the initiating stroke or disease [5].

According to The Kidney Disease Improving Global Outcomes (KDIGO) initiative, if any structure or function violations of the kidneys persist for more than 3 months, the patient can be called a CKD patient [6]. KDIGO presents a severity categorization that describes the different stages of CKD based on glomerular filtration rate (GFR; either estimated (eGFR) or measured (mGFR)) and albuminuria degree. GFR is an established marker of excretory kidney function, and albuminuria, in turn, is an indicator of a violation of the renal barrier (damage to the glomeruli). Both GFR and albuminuria are used to classify CKD and have been declared reliable predictors of long-term chronic kidney disease outcomes [7].

Since the kidney consists of numerous independent functional and anatomical “units” (nephrons), GFR can be expressed by the equation: GFR (total) = GFR (single nephron) × the number of nephrons, taking into account that the GFR (single nephron) represents the filtering potential of specific nephrons [8]. This particular equation means that if the number of nephrons is lower, the total GFR does not seem to change as long as the remaining nephrons can elevate their contribution. However, total GFR volume lowering means an essential loss of nephrons, while the remaining nephrons can function at the maximum possible GFR (single nephron) [9].

Thus, CKD represents a loss of the number of nephrons. In addition, the KDIGO categories describe the risk of developing renal failure, e.g., end-stage renal failure (ESRD), which requires renal replacement therapy (peritoneal dialysis, hemodialysis, or kidney transplantation), and several other unfavorable consequences, such as the risk of cardiovascular disease (CVD), acute kidney injury (AKI), infection, hospitalization, and death. The KDIGO categorization has confirmed its effectiveness in making decisions on patient management, but contradictions still exist [6].

Although the classification of CKD severity by GFR and albuminuria is very valuable, the detection of risk factors for CKD plays an important role in the best management and is recommended by current guidelines [10]. Multiple complications, such as anemia, metabolic acidosis (decreased acid excretion by the kidneys), and cardiovascular diseases, are linked with chronic kidney disease and cause difficulties in patient management [11].

Renal functional reserve (RFR) is the ability of the kidney to enhance the glomerular filtration rate in response to physiological or pathological stimuli or conditions. RFR in clinical practice is determined as the difference between peak ‘stress’ GFR induced by the test and the baseline GFR. When hyperfiltration occurs, RFR may be fully or partially used to achieve normal or supranormal renal function [12]. Classic markers of renal functions (i.e., GFR) can maintain normal ranges up to 50% nephron loss, in contrast to the RFR test, which can represent a sensitive and early way to assess the functional decline in the kidney. Thus, a reduction in RFR may represent the equivalent of renal frailty or susceptibility to insults [13].

More precise prediction of all-cause mortality and fatal/non-fatal CVD was achieved by the calculation of GFR on the base of cystatin C. EGFRcys and albuminuria are independent risk factors for fatal/non-fatal CVD and should be considered in cardiovascular risk prediction to advise primary preventative treatment decisions [14]. Together with urine albumin–creatinine ratio, which is the marker of albuminuria, eGFRcys was used to create heat maps for the prediction of all-cause mortality, composite fatal/non-fatal cardiovascular disease (CVD), fatal CVD, and end-stage kidney disease. Measuring serum cystatin C is the test that should be used, and eGFRcys can be beneficial for CKD diagnosis and CVD prediction. This can allow the thus making of important clinical decisions around the implementation of CVD risk-lowering therapies in addition to conventional CVD risk factor calculators [15].

## 3. Cardiovascular Risk in CKD

**Figure 1:**
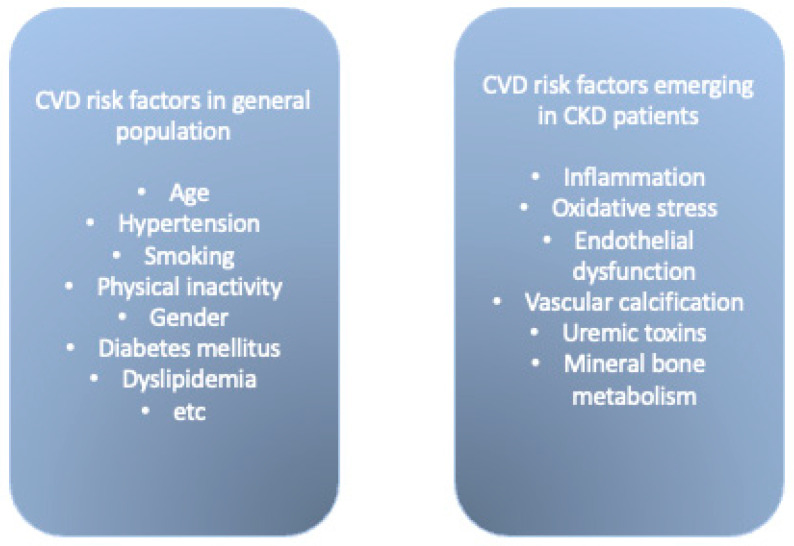
CVD risk factors in general population and in CKD patients.

**Figure 2:**
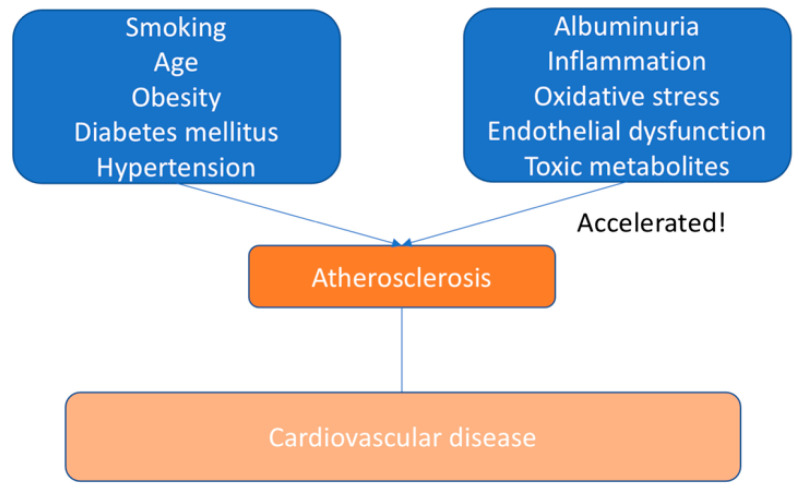
Additional risk factors related to CKD, which lead to atherosclerosis development.

The main causes of cardiovascular morbidity and mortality do not have the same predictive significance in patients with CKD, especially with progressive CKD, as in the general population [3]. This indicates that additional pathogenic processes are possible, which could be reflected in emerging risk factors associated with CKD. We summarized risk factors for CVD in the general population and CKD patients in Figure 1 and Figure 2.

Nevertheless, the specific molecular pathways activated by associated with CKD factors are characterized insufficiently. Regardless of these hypotheses, in contrast to patients with normal renal function, elderly patients with CKD had an extremely small number of genes differentially expressed in uninfected arteries [16]. Moreover, among the genes sequentially modulated in vascular smooth muscle cells (VSMCs), the expression of only twenty-three genes was lowered, and the expression of eight was elevated. Due to uremia, mRNA expression and the protein content of the alpha subunit of the hypoxia-inducible factor 3 were increased. However, the content of vimentin increased with a decrease in its mRNA expression [17,18].

### 3.1. Arterial Hypertension

Generally, in most patients, arterial hypertension is not a cause but a consequence of chronic kidney disease [19]. Yet, at the earliest stages of CKD, hypertension may be present and well documented, which favors cardiovascular morbidity and mortality. Arterial hypertension is very common in children with CKD (54–70% of patients) [20]. Hypertension is the result of triggering the neurohumoral axis (specifically the activity of catecholamines and aldosterone), activation of RAS, and hypervolemia. In some circumstances, hypertension occurs due to drugs used to treat the underlying kidney disease—corticosteroids or calcineurin inhibitors. The crucial element of CKD treatment for the prevention of cardiovascular diseases is the control of arterial hypertension [21].

### 3.2. Dyslipidaemia

In patients with chronic kidney disease, lipid metabolism disorders and modifications of lipid particles are usually associated with the uremic toxin, which favors the development of atherogenesis. Posttranslational modifications of lipid particles associated with CKD suggest pro-inflammatory effects and endothelial dysfunction. The comparative contribution of specific anomalies to the accelerated development of cardiovascular diseases in patients with chronic kidney disease has yet to be determined [22,23].

The quantitative changes in serum lipids are not particularly proatherogenic in CKD. Thus, triglyceride levels were associated with subclinical atherosclerosis in CKD G3, whereas in CKD G4-5, only total cholesterol showed a weak association with the plaque presence. Moreover, low-density lipoprotein (LDL)-cholesterol appeared to be not so effective in coronary risk prediction in patients with CKD [24]. Qualitative changes in lipid profile, on the other hand, were found to be associated with a higher atherogenic profile. In this regard, CKD is characterized by an accumulation of VLDL (very-low-density lipoprotein) particles, a reduction of LDL particle size, and changes in the cholesterol and triglyceride content in LDL and HDL (high-density lipoproteins, which gain triglycerides and lose cholesterol). Notably, HDL in CKD patients turned out to lose its atheroprotective properties and even harm the vasculature [25].

### 3.3. Mineral Bone Metabolism

The mineral bone metabolism and its changes were shown to contribute to the vascular injury accompanying CKD. However, the relation of medial vascular calcification often observed in patients with CKD to the atherosclerotic process remains controversial. These doubts are supported by the medial calcification of small arteries, which are rich in VSMCs and where calcification is typically observed without plaques, in contrast to big arteries [26].

The hallmark of CKD–mineral and bone disorder (CKD-MBD) is vascular calcification, which occurs early in the CKD course. The basis for this process is the instability of calcium and phosphate ions in the circulation plus the abnormal differentiation of VSMC to osteoblast/chondroblast-like cells. Moreover, important contributors to the CKD-MBD in more advanced stages of CRD are phosphate and calcitriol, which can directly modify the phenotype of VSMCs. Calcitriol acts in a dose-dependent manner and is modulated via an interplay with calcium and phosphate availability and other environmental factors. Both a direct calcitriol-driven increase in VSMC calcification and the opposite effect has been described [27].

NEFRONA study (Observatorio Nacional de Atherosclerosis en NEFrologia) revealed that higher serum phosphate levels were independently linked to plaque prevalence in every investigated CKD category (G3, G4, and G5D) and higher hsCRP (high-sensitivity C-reactive protein) emerged in G4-G5 and lower 25(OH)D levels in G5D. At the same time, indirect methods (ABI) of assessment of peripheral artery disease led to the same results [28]. This allows us to suggest that the direct assessment of atherosclerosis in the form of plaques can be of particular importance. Another mechanism of vascular calcification implies calcium storage in the intima layer of the arterial wall. An endothelial cell can also be modified by CKD, but its phenotype cannot be switched to osteoblast-like [29].

### 3.4. Inflammation

Inflammation is an essential part of atherosclerosis and CKD pathogenesis. Enhanced levels of CRP and pro-inflammatory cytokines, increased reactive oxygen species (ROS) generation, and an activated phenotype of circulating monocytes and resident cells are linked to the pro-inflammatory profile of the advanced renal disease. The pro-inflammatory landscape in CKD can depend not only on enhanced synthesis but also on reduced clearance of mediators of inflammation. Thus, calcium-phosphate crystals induce a pro-inflammatory response in macrophages. Uremic toxins should be mentioned as another pro-inflammatory driver. Atherogenesis can be promoted through the pro-inflammatory responses in macrophages and vascular and parenchymal cells resulting from protein-bound uremic toxins generated from gut microbiota metabolites, such as p-cresyl-sulfate and indoxyl sulfate [30].

### 3.5. Proteinuria

Severe proteinuria was shown to be associated with an atherogenic lipid profile. Such a profile contains high levels of lipoprotein A and LDL cholesterol. Additionally, microalbuminuria and recently termed A2 albuminuria are linked to an increased risk of cardiovascular mortality and non-fatal cardiovascular events. This was shown independently from the GFR influence. However, an exact mechanism linking albuminuria to CVD has not been found yet. It is important to note that albumin can be observed in the urine only when the reabsorptive capacity of proximal tubular cells has been exceeded. Thus, even mild albuminuria is associated with proximal tubular cells overloaded with filtered proteins. This leads to tubular cell stress, decreases Klotho production, and triggers an inflammatory response [31].

## 4. Cardiovascular Diseases

The high frequency of cardiovascular diseases in CKD patients may be related to a fairly high prevalence of hypertension, dyslipidemia, hyperuricemia, glucose metabolism disorders, obesity, systemic inflammation, and oxidative stress [32]. People with CKD aged from 25 to 34 years have at least 100 times higher risk of mortality from cardiovascular diseases in contrast to the general population [33].

Cardiovascular changes associated with CKD are similar to the accelerated aging process related to a shortening of telomere length. The underlying mechanisms of accelerated calcification of blood vessels and the heart found in the CCD and ESRD are not sufficiently investigated. In the early stages of CKD (CKD G1–G2), atherosclerotic processes dominate (e.g., invasion of macrophages, plaque formation, and thickening of the arterial wall) [34]. During the development of CKD, inflammatory factors and calcification of media contribute to the degeneration of the vascular wall. The various factors involved cause changes in the profile of risk factors and have different effects on outcomes during CKD [35]. Hypertrophy of the left ventricle is also possible (either concentric (in the case of arterial hypertension) or eccentric (in the case of hypervolemia and anemia)), as well as dilation, which results in systolic and diastolic dysfunction. It was demonstrated that the cause of left ventricular hypertrophy in CKD is early and prolonged induction of fibroblast growth factor 23 (FGF23) [36].

In patients with CKD who have not started renal replacement therapy, the risk of cardiovascular events is as high as in people with diagnosed coronary artery disease. Insulin resistance, high blood pressure, vascular calcification, inflammation, and a decrease in protein-energy levels increase risks [37]. Myocardial stunning is a phenomenon in which transient episodes of ischemia are observed. These episodes can be a result of hemodialysis, which has a direct adverse effect on the heart. Unlike the general population, patients with low GFR or patients on hemodialysis have increased mortality due to adverse cardiovascular events. Thus, CVD risk factors should be intensively managed at all stages [38].

## 5. Determining Atherosclerosis in CKD

The determination of atherosclerosis is the main question. Earlier, it was described as a hypothetical atherosclerotic event such as myocardial infarction [39]. This definition has a significant disadvantage since during the sessions of peri-hemodialysis, arrhythmia may occur, leading subsequently to sudden cardiac death, which is considered the main cause of cardiovascular mortality. It is noteworthy that if sudden cardiac death due to the cause of primary arrhythmia happens outside the hospital, it is difficult to distinguish it from death from myocardial infarction [40]. An alternative functional approach may define atherosclerosis as events that can be avoided with the assistance of statin therapy. This definition focuses on clinically significant episodes that indicate a causal relationship as the response to targeted interventions is studied [41].

To determine the effect of atherosclerosis in CKD, randomized clinical trials (RCTs) targeting pro-atherosclerosis factors, in particular, high LDL cholesterol (low-density lipoproteins), are needed [42,43]. However, this approach has been tested, and the trials keep the same doubts. These doubts are related to the question of whether the failure of such tests indicates different pathogenesis of cardiovascular disease in CKD or the existence of a point-of-no-return. The morphological definition of subclinical atherosclerosis can also be used. It is based on an elevation in the thickness of intima media (IMT), on the presence of plaques during ultrasound examination of the carotid arteries and femur or computed tomography, confirming coronary calcification [44]. Difficulties with this definition include elevated IMT unrelated to atherosclerosis (as reported, for example, for Fabry’s disease), coronary calcification unrelated to atherosclerosis, and decreased clinical significance to event-based definitions. A significant amount of imaging data was received due to the evaluation of coronary calcification using computed tomography [45]. Even though these studies imply a higher burden on atherosclerosis in patients with CKD in the general population, autopsy studies report that in developing CKD, calcification sites could be located in the media layer, which is not typical for atherosclerosis in the general population. These autopsy reports coincide with the widespread use of calcium-based phosphate binders and high doses of calcitriol [46]. It is necessary to conduct large population studies comparing CKD with patients who do not suffer from CKD to assess the relative prevalence of atherosclerosis in CKD using morphological determination. The advantage of this definition is that for clinical trials of measures aimed at combating atherosclerosis, it is possible to select patients who have already developed atherosclerosis, while the current approach assumes the appointment of measures aimed at combating atherosclerosis based on risk indicators for the population [47]. The current approach could result in the appointment of measures aimed at the therapy of atherosclerosis to those patients who do not suffer from this disease. Therefore, the result of this intervention will not benefit. At the level of clinical trials, modern approaches may hinder the ability to quantify the actual benefits of drugs that persistently lead to manifestations of atherosclerosis in people suffering from this inflammatory disease. Since the major problem of atherosclerosis associated with CKD is the insufficiency of RCTs with positive results, it was decided to focus on atherosclerosis, which is defined as the presence of plaques as a potential approach to eliminating existing limitations [48].

## 6. Pathogenesis of Atherosclerosis

Under normal conditions, endothelial cells of the arterial wall resist leukocyte adhesion and aggregation. When the endothelium is damaged due to cigarette smoking or hypertension, LDL molecules infiltrate the arterial wall and are oxidized by intima enzymes. The consequences of this process are activation of endothelial cells and elevated regulation of several types of leukocyte adhesion molecules [49,50]. These molecules give leukocytes along the vascular surface the opportunity to adhere to the activation site. It was revealed that the vascular cell adhesion molecule-1 (VCAM-1) is of key importance in this process. Monocytes and lymphocytes most often bind to VCAM-1 [51]. After fixation, chemokines produced in the underlying intima promote the migration of these cells through the intraendothelial junctions and into the subendothelial space. Nowadays, the chemoattractant cytokine monocyte chemoattractant protein-1 is recognized as a key factor that favors the migration of monocytes. It interacts with the C-C chemokine receptor type 2 (CCR2) monocytes by recruiting monocytes into the arterial endothelium and facilitates their penetration between endothelial cells by diapedesis [52,53].

Once in the subintimal space, under the action of a macrophage colony-stimulating factor, monocytes differentiate into macrophages. In turn, macrophages express scavenger receptors that promote the absorption of modified lipoprotein particles. The cytoplasm is imbued with lipid particles, giving macrophages a typical microscopic appearance of foam cells that can be detected in atherosclerotic lesions [54].

In addition, macrophages express Toll-like receptors. In comparison with scavenger receptors, Toll-like receptors can trigger a signaling cascade that results in cell activation. Activated macrophage produces inflammatory cytokines, proteases, and cytotoxic types of oxygen and nitrogen radicals [55]. Oxidized LDL and heat shock proteins are ones of recognized activators of these receptors. Cells in atherosclerotic lesions show a spectrum of Toll-like receptors, and plaque inflammation could partly depend on this pathway. There are relatively small populations of T-lymphocytes in the atheroma [56]. Despite the low content of white blood cells in plaques, these cells of the adaptive immune response have an essential regulatory effect on more abundant monocytes. Within the plaque, T cells exhibit heterogeneity of functions. Some subsets are pro-inflammatory (T helper cells 1), while others tend to suppress inflammation (T helper cells 2) [57].

The atherosclerotic lesion contains cytokines that induce the T-helper 1 reaction. T-helper 1 cells produce interferon-γ, which triggers macrophages and enhances the synthesis of inflammatory cytokine tumor necrosis factor and interleukin (IL) 1 [58]. These cytokines enable the production of multiple inflammatory and cytotoxic molecules in macrophages and vascular cells, which only aggravate the inflammatory process. It is important to note that T helper 2 cells not only produce antiatherosclerotic cytokines but also favor elastolysis, which can cause aneurysm formation. Thus, regardless of whether there is a predominant T-helper 1 or T-helper 2 response in the plaque, the vascular disease can still develop. However, it is also worth paying attention to the fact that its phenotype may differ [59,60,61].

## 7. Managing ASCVD in CKD Populations

Medication and interventional therapy are used for the general population and individuals with chronic kidney disease. Moreover, with these interventions, kidney transplantation reduces the risk of cardiovascular events in individuals with chronic kidney disease. The evidence of these interventions in atherosclerotic cardiovascular disease in chronic kidney disease is presented [62].

### Medical Management of Stable Ischemic Heart Disease in CKD

Advances in the treatment of stable coronary artery disease (SIHD) have not only led to lifestyle changes but also resulted in significant reductions in morbidity and mortality in the general population. These results were achieved with the contribution of drugs such as 3-hydroxy-3-methyl-glutaryl-coenzyme, reductase inhibitors (i.e., statins), and aspirin [63]. Statins have been extensively studied in chronic kidney disease populations. It is worth noting that these studies included people selected based on kidney function and are not based on the presence of SIHD [64].

Based on three large-scale studies (Die Deutsche Diabetes Dialysis, An Assessment of Survival and Cardiovascular Events, and Study of Heart and Renal Protection), it was concluded that neither primary nor secondary prevention using statins in patients with end-stage renal disease demonstrated benefits [65]. In the Study of Heart and Renal Protection, it was noticed that in patients with CKD who are not on dialysis and who took drugs such as simvastatin plus ezetimibe, major adverse cardiac events (MACE) decrease. Despite this, however, there were no differences in the outcome of mortality [66]. Data from large general population studies have demonstrated that meta-analysis of patients with CKD indicates a decrease in mortality and cardiovascular events. Thus, based on the Kidney Disease: Improving Global Outcomes (KDIGO), lipid guidelines recommend prescribing statin therapy to all individuals with CKD older than fifty years and those at high risk of developing cardiovascular diseases aged 18–49 years [62].

Altered platelet reactivity is a common trait of individuals with chronic kidney disease. Existing data indicate that patients with CKD have an elevated platelet reactivity to adenosine diphosphate in contrast to patients with normal renal function. On the other hand, some studies have shown that patients with CKD are more prone to bleeding [67]. Concerning SIHD in patients with CKD, this has two crucial meanings: (1) the role of antiplatelet therapy, specifically aspirin, for the primary prevention of coronary artery disease in individuals with CKD is limited to the prevention of myocardial infarction, but not mortality; (2) progress in the medical treatment of coronary heart disease in the general population consists in the presence of powerful antiplatelet regimens to maintain the patency of the stent after percutaneous coronary intervention (PCI) [68].

The limitations of the effectiveness of P2Y12 inhibitors and aspirin in CKD seem to be a serious factor contributing to the decrease in benefits after percutaneous coronary intervention (PCI) in people with CKD. It is especially important because in-stent thrombosis is quite a big problem in people with CKD, especially in those with end-stage renal disease (ESRD) [69].

## 8. Pathophysiologic Considerations of ASCVD in CKD

In the presence of coronary artery disease, atherosclerotic plaque becomes a therapeutic target. Revascularization of critical lesions associated with acute coronary syndrome (ACS), as well as stabilization of atherosclerotic plaques through treatment with drugs such as statins, remain the primary basis of therapy [70]. Despite this, in terms of decreasing MACE in patients with CKD, these therapeutic agents have provided incomplete benefits. Some observations, several of which were described above, show that a set of more complex factors contributes to atherosclerotic cardiovascular disease development in patients with CKD [3].

First of all, with CKD, calcification of the atherosclerotic plaque speeds up. However, it also increases in the medial layer of the arteries, which was not observed in patients with preserved kidney function. The cause of vascular calcification, especially medial calcification, is secondary hyperparathyroidism of renal origin. A study by Moe et al. showed that in patients with developing chronic kidney disease, calcification in the samples of the lower epigastric artery is medial [71]. This was revealed during a spiral computed tomography, which positively correlated with serum phosphorus levels and with the total content of calcium and phosphorus in serum [72]. Kono et al. found a positive correlation between the level of phosphorus in the blood serum and the density of calcification of coronary atherosclerotic plaques, which was identified using intravascular ultrasound [73]. A study by Ganesh et al. demonstrated that in hemodialysis patients, increased levels of hydroxyapatite are associated with a high risk of sudden death [74].

These pieces of evidence directly state several important points: (1) calcification of the vascular system is part of the uremic pathological process, and (2) hyperparathyroidism of renal origin is involved in the process that stimulates calcification of media and plaques in CKD [75]. The coronary arterial system calcification results in high stimulation of macrophages and the subsequent release of inflammatory mediators, such as tumor necrosis factor-α. In experimental models, calcification is also associated with the death of vascular smooth muscle cells, which is able to result in impaired vascular reactivity or increased plaque rupture [76]. In the study by Takx et al., a clinical correlation was shown, which demonstrated increased absorption in the arterial system (i.e., ascending aorta) of ^18^fludeoxyglucose during CT positron emission tomography [77]. On this basis, it can be concluded that there is an immune-mediated inflammatory activity in individuals with chronic kidney disease in contrast to comparable control groups without CKD. High activity did not depend on the calcium score in the coronary arteries [78]. According to studies by Nadra et al. [79] and Ewence et al. [80], no comparison was made between aortic calcification sites and indicator absorption to indicate calcification areas by sites of greater indicator absorption.

The growth of inflammation in the arterial wall in individuals with chronic kidney disease is indisputable evidence. Several different authors proposed proof showing that control of the calcium intake or lowering parathyroid hormone levels, in particular with the help of calcimimetic cinacalcet, results in a reduction in calcium content in the vasculature of individuals with end-stage renal disease. Eventually, this did not result in a decrease in atherosclerotic cardiovascular disease in patients with end-stage renal disease [81].

Coronary microcirculation (i.e., arterioles and capillaries) is another aspect of coronary circulation that receives more and more attention from investigators [82]. Several indicators are used to assess coronary circulation: fractional flow reserve, coronary flow reserve, and microvascular resistance index. Microcirculation dysfunction was associated with negative outcomes in patients without chronic kidney disease [83].

Coronary microvascular dysfunction correlates with atherosclerotic cardiovascular disease in chronic kidney disease, namely with false positive perfusion scintigraphy of the myocardium and muted clinical benefits for revascularization of the coronary arteries [84].

Some researchers have studied the direct measurement of the parameters of coronary microcirculation. However, until now, the methods had differences. For example, both direct angiography and PET were applied to assess the reserve of coronary bloodstream in individuals with chronic kidney disease [85]. In both cases of lower GFR, the reserve of the coronary bloodstream was lower. To date, it is clear that the clinical application of this parameter has yet to be studied in depth. Factors affecting endothelial function (for example, insulin) capable of metabolic parameters of bone tissue to cause an improvement of coronary microvascular function in individuals with chronic kidney disease remains unclear. Usually, patients with developing ASCVD also have risk factors for cardiovascular disease, including albuminuria and general growth in inflammatory markers [86,87,88]. In their study, Rein et al. showed that the existence of albuminuria is linked with a higher probability of detecting significant coronary artery stenosis (≥ 50%) during angiography. A direct relationship was also established between the amount of albuminuria and the number of vessels with significant stenosis [89].

In another independent analysis, Rein et al. evaluated albuminuria as an important predictor of future major adverse cardiac events, comparing patients without known coronary artery disease but with albuminuria and patients with a known history of coronary artery disease but without albuminuria [90]. Thus, these authors state that albuminuria is equivalent to the risk of coronary artery disease (CAD). It was found that inflammation caused by changes in lipid metabolism (namely, increased levels of oxidized LDL, as well as low and poorly functioning levels of HDL) is also associated with cardiac risk in patients with chronic kidney disease [91].

Myocardial fibrosis is common among people with chronic kidney disease and end-stage renal disease. In kidney disease, cardiac fibrosis consists of intermyocytic fibrosis, which results in a reduction in the ratio of capillaries to myocytes. Mediators identified as important in chronic kidney disease-related cardiac fibrosis include elevated fibroblast growth factor-23, lowered Klotho levels, and elevated phosphorus levels [92]. Ultimately, most of these mediators resulted in potentiation of myocardial fibrosis induced by the transforming growth factor β1. It is noteworthy that cardiac fibrosis is also the result of myocardial ischemia. This indicates that the environment of chronic kidney disease causes changes in the myocardium similar to ischemia. Nevertheless, it seems that due to a decrease in the density of capillaries in the myocardium, these changes themselves favor further ischemic changes. The combination of myocardial fibrosis and coronary microvascular dysfunction results in repeated ischemic events favoring myocardial dysfunction [93].

In the end, kidney transplantation, or rather correction of the uremic environment, results in a much greater decrease in clinical cases of cardiovascular diseases in comparison with revascularization of the coronary arteries [94]. This applies to numerous pathophysiological processes, the correction of which requires functioning nephrons. The inflammation linked to uremia is also corrected and could be partially linked to a decrease in cases of atherosclerotic cardiovascular disease after transplantation. It is extremely important to take into account that poorly controlled parameters of CKD and retransplantation of inflammation associated with uremia resulted in deterioration of cardiovascular outcomes after transplantation, emphasizing the importance of pretransplantation for the control of CKD [95].

## 9. Conclusions

It was shown that in the general population with preserved kidney function, cardiovascular diseases are best prevented by minimizing the risk of atherosclerosis. In end-stage renal failure and chronic kidney disease, the atherosclerotic plaque turns out to be a smaller element of the overall burden of CVD. The main components of cardiovascular diseases in patients with CKD are extensive calcification of blood vessels and plaques, as well as endothelial dysfunction. This has a curious and unusual connection with the uremic environment since transplantation lowers the observed MACE compared to patients on hemodialysis or peritoneal dialysis. The approach to this type of atherosclerotic cardiovascular disease was complex, and the currently available treatment methods have not resulted in a significant decrease in ASCVD in individuals with chronic kidney disease, except statins in patients with CKD before dialysis. Nowadays, the presented data force us to raise questions about the expediency of revascularization of coronary arteries in people with CKD and CAD.

## Data Availability

Not applicable.
